# The interplay of goal-driven and stimulus-driven influences on spatial orienting

**DOI:** 10.3758/s13414-016-1121-8

**Published:** 2016-06-03

**Authors:** Mara Otten, Daniel Schreij, Sander A. Los

**Affiliations:** 1 Universiteit Utrecht, Utrecht, The Netherlands; 2 Vrije Universiteit Amsterdam, Amsterdam, The Netherlands; 3 Department of Cognitive Psychology, Vrije Universiteit, Van der Boechorststraat 1, 1081 BT Amsterdam, The Netherlands

**Keywords:** Attentional capture, Eye movements, Visual attention

## Abstract

Search for a target stimulus among distractors is subject to both goal-driven and stimulus-driven influences. Variables that selectively modify these influences have shown strong interaction effects on saccade trajectories toward the target, suggesting the involvement of a shared spatial orienting mechanism. However, subsequent manual response times (RTs) have revealed additive effects, suggesting that different mechanisms are involved. In the present study, we tested the hypothesis that an interaction for RTs is obscured by preceding multisaccade trajectories, promoted by the continuous presence of distractors in the display. In two experiments, we compared a condition in which distractors were removed soon after the presentation of the search display to a standard condition in which distractors were not removed. The results showed additive goal-driven and stimulus-driven effects on RTs in the standard condition, but an interaction when distractors were removed. These findings support the view that both variables influence a shared spatial orienting mechanism.

Two fundamentally different mechanisms are said to control visual spatial attention. When attention is allocated to an object because of its salience, it is called *bottom*-*up* or *stimulus*-*driven* attentional selection (Franconeri, Simons, & Junge, 2004; Theeuwes, 1991, 1992, 2010; Yantis, 1993; Yantis & Johnson, 1990a, 1990b; Yantis & Jonides, 1990). *Top*-*down* attentional selection, on the other hand, depends on the goals of the participant (Bacon & Egeth, 1994; Folk & Remington, 1998; Folk, Remington, & Johnston, 1992; Folk, Remington, & Wright, 1994; Gibson & Kelsey, 1998; Theeuwes, 2010; Wolfe, 1994). There have been extensive debates about whether the capture of spatial attention is always contingent on top-down attentional control settings (Folk et al., 1992), as posited by the contingent attentional-capture theory (see also Folk & Remington, 1998, 2006; Folk, Remington, & Wu, 2009; Gibson & Amelio, 2000; Pratt, Sekuler, & McAuliffe, 2001), or whether some salient objects are able to override these attentional settings and thus capture attention in a purely bottom-up fashion (Theeuwes, 1994a).

A stimulus that is known to elicit a strong bottom-up signal is the so-called *abrupt onset*: a stimulus that suddenly appears somewhere in the visual field in an empty space. It has often been demonstrated that this salient stimulus is involuntarily prioritized in search (Yantis & Hillstrom, 1994; Yantis & Jonides, 1984, 1990), leading to a speeded response if it happens to be the target. In turn, an onset can cause interference, leading to a slowed response when another object is the target of search, even when the onset distractor is completely irrelevant to the observer’s goal. These properties therefore suggest that an onset stimulus is able to involuntarily capture one’s attention (Belopolsky, Theeuwes, & Kramer, 2005; Franconeri et al., 2004; Jonides & Yantis, 1988; Theeuwes, 1994a, 1994b; Yantis, 1993; Yantis & Jonides, 1984).

By contrast, Folk et al. (1992) claimed that an onset (or, in fact, any stimulus) can only capture attention if it possesses a property that the observer is actively searching for. To support this claim, they instructed observers to make a speeded response to the identity of a red target character (“X” or “=”), which appeared within one of four placeholder boxes around a fixation cross. The target display was briefly preceded by one of two types of cue displays, which were both valid only at chance level with respect to the location of the upcoming target. In the *color*-*cue* display, four dots were briefly flashed around each placeholder box. Around three of the placeholder boxes these dots were colored white, but the fourth box was surrounded by *red* dots—the same color that defined the impending target stimulus. In the *onset*-*cue* display, only one box was briefly surrounded by four *white* dots—lacking any defining feature of the impending target stimulus. Folk et al. (1992) showed that there was an effect of cue validity on response times (RTs) in the color-cue but not in the onset-cue condition. On the basis of this and similar findings, they argued that a valid cue has a facilitating effect only when it shares defining properties with the target (in this case, the color red). This idea is referred to as *contingent* attention capture.

Schreij, Owens, and Theeuwes (2008) used a modified version of the spatial-cuing paradigm of Folk et al. (1992) to further investigate the interaction between contingent precues and noncontingent onsets. Essentially, they combined Folk et al.’s (1992) “contingent” cue validity manipulation with Theeuwes’s (1992) “noncontingent” onset presence manipulation. In particular, each trial started with a cue display, in which a red cue (four dots) predicted the location of a red target stimulus at chance level. Subsequently, on half of the trials, an onset was added to the target display (the “onset-present condition”), whereas on the other half of the trials, no onset was added to the target display (the “onset-absent condition”). The onset stimulus (in the onset-present condition) was an additional white placeholder box, containing a white distractor stimulus, between any two of the already present placeholder boxes. Thus, instead of presenting the onset distractor as a precue consisting of four white dots, they presented the onset distractor as a new object among the placeholders that were present from the beginning of the trial. The results showed that manual RTs were shorter when the red cue correctly indicated the location of the target, suggesting that the cue captured attention in a contingent fashion, thereby replicating the findings of Folk et al. (1992). However, the presence of the abrupt onset also slowed observers’ responses, regardless of whether the cue was valid or invalid. Since the onset was not part of the attentional set of the participants, Schreij et al. (2008) considered this finding incompatible with the contingent attentional capture theory. The delay in RTs led the authors to conclude that the abrupt onset captured attention independently of the top-down settings of the participants, and that spatial attention was allocated to the abrupt onset.

However, Folk et al. (2009) gave a different interpretation of the findings reported by Schreij et al. (2008). If both the cue and the onset captured attention, as claimed by Schreij et al. (2008), then one would expect to observe an interaction between cue validity and onset presence. In particular, when an onset was present, it would vie with the cue for attentional control. So, the cue would less often attract attention when an onset was present than when it was absent, which should result in a reduced effect of cue validity. However, instead of such an underadditive relationship, Schreij and colleagues (Schreij et al., 2008; Schreij, Theeuwes, & Olivers, 2010a, 2010b) consistently found additive effects of onset presence and cue validity (see also Folk et al., 2009; Wu, Remington, & Folk, 2014), which suggests that the underlying mechanisms for stimulus-driven and contingent capture are independent. This reasoning led Folk et al. (2009) to propose that the interference caused by the abrupt onset reflects nonspatial filtering costs (Kahneman, Treisman, & Burkell, 1983), rather than a spatial orienting response of attention. A filtering operation would be necessary to disregard and suppress the onset as a potential target candidate before attention could be allocated to the real target. Assuming that a filtering operation preceded the allocation of attention, and that these processes were influenced by onset presence and cue validity, respectively, the additive pattern of these factors would be the necessary consequence according to the classic additive-factor logic (Sternberg, 1969).

To gain greater insight into the cause of the robust additive relationship between cue validity and onset presence, Schreij, Los, Theeuwes, Enns, and Olivers (2014) reused the paradigm of Schreij et al. (2008) and recorded eye movements in addition to manual RTs. It is generally accepted that an eye movement is preceded by a shift in spatial attention to its destination (Awh, Armstrong, & Moore, 2006; Deubel & Schneider, 1996; Godijn & Theeuwes, 2002; Hoffman & Subramaniam, 1995; Peterson, Kramer, & Irwin, 2004; Rizzolatti, Riggio, Dascola, & Umiltà, 1987), so eye movement trajectories toward the target stimulus provide an overt expression of the time course of attention during a trial. This allowed Schreij et al. (2014) to examine the role of attention in the origin of the additive pattern in RTs.

Schreij et al. (2014) found strong interaction effects between cue validity and onset presence on saccade trajectories, suggesting that the onset elicited a spatial orienting response, just as the contingent cue did. In fact, the relative frequencies of first saccades (from central fixation to any one object in the display) precisely revealed the underadditive pattern envisaged by Folk et al. (2009): In the onset-absent condition, the eyes went substantially more often to the cue than in the onset-present condition, clearly suggesting that the cue and the onset vie for attentional control. In contrast, the mean manual RT data again showed additive effects of cue validity and onset presence, thereby replicating earlier studies. A full analysis of the eye movement trajectories toward the target stimulus revealed why the underadditive pattern observed in the relative frequency of first saccades did not propagate to the mean RTs. In both the onset-present, valid-cue condition and the onset-absent, invalid-cue condition, the eyes reached the target in a maximum of two saccades on almost every trial. However, in the onset-present, invalid-cue condition, there were also trajectories that included three saccades before the target was reached: from fixation via the cue to the onset, and only then to the target, or from fixation via the onset to the cue, and only then to the target. These three-saccade trajectories lengthened the time it took the eyes to finally arrive at the target. Together, the presence of an onset decreased the proportion of saccades to the invalid cue, but at the same time increased the mean duration of the saccade trajectory to the target, in view of the occasional three-saccade trajectories. As it turned out, these opposite influences balanced out, resulting in the additive pattern of cue validity and onset presence on manual mean RTs. This analysis led Schreij et al. (2014) to conclude that the observed additivity in RTs from earlier studies (Schreij et al., 2008; Schreij et al., 2010a, 2010b) was not a consequence of nonspatial filtering effects brought about by the onset (Folk et al., 2009), but rather reflected that the onset can direct attention to its location even after attention has first been captured by an invalid cue.

A direct corollary of the eye movement dynamics observed by Schreij et al. (2014) is that an underadditive interaction on mean RTs should be observed if all trials with three-saccade trajectories were eliminated. In the present study, we attempted to eliminate such three-saccade trajectories by means of an experimental manipulation. We used the paradigm of Schreij et al. (2014), but added one condition: On half of the trials, all objects except the target object disappeared during the execution of the first saccade. In this *remove* condition, the target was the only object left after the first saccade, leaving the eyes nowhere else to go to. If successful, this manipulation should lead to an underadditive interaction between cue validity and onset presence on mean RTs, thereby supporting the conclusion of Schreij et al. (2014) that both the cue and the onset capture attention by a common spatial orienting mechanism. An additive relationship was expected to be found again when all objects remained on display until the end of the trial, which would be in line with the previous findings (Schreij et al., 2008; Schreij et al., 2010a, 2010b). In an additional experiment, we verified whether the same pattern of RTs would be observed if the task was performed without making saccades.

## Experiment 1

### Method

#### Participants

Twenty-four students took part in this experiment in exchange for money. The participants, two men and 22 women, ranged in age from 21 to 32 years (*M* = 24.4), and all reported normal or corrected-to-normal vision and no color blindness.

#### Apparatus

The experiment was run on a PC in a dimly-lit room. Stimuli were presented on a 19-in. CRT monitor (1,024 × 768 pixels). Participants were seated approximately 75 cm from the screen with their head on a chinrest and their index fingers resting on top of the “N” and “M” keys of a qwerty keyboard. The OpenSesame experiment builder (Mathôt, Schreij, & Theeuwes, 2012) was used for presentation of the stimuli and response recording. Eye movements were recorded with EyeLink 2000 (SR Research), a video-based eyetracker with a sampling rate of 2 kHz.

#### Stimuli

**Fig. 1:**
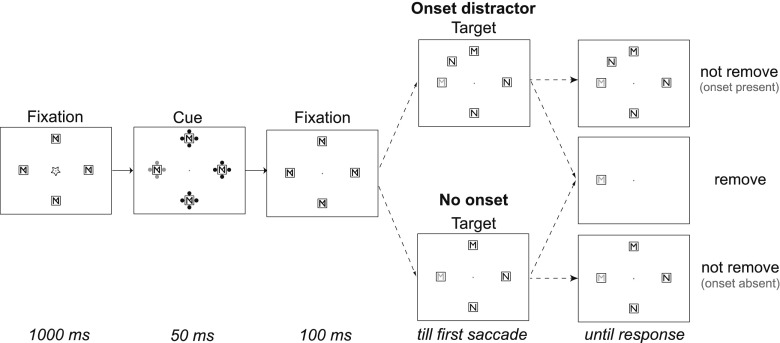
Illustration of the time courses of four trial types in the valid-cue condition. The target display (following the second fixation display) either contained an onset or no onset, and during the subsequent first saccade, the distractor objects were either not removed or removed. The stimuli are shown in opposing contrasts, with red stimuli shown in gray; the displays are not drawn to scale

The stimulus displays were presented on a black background. There were three types of displays: a fixation display, a cue display, and a target display (see Fig. 1). The fixation displays consisted of a white, 0.07° fixation dot [CIE(0.286, 0.311), 100 cd/m^2^] in the center of the screen, surrounded by four white placeholder boxes (0.7° wide) above, below, to the left, and to the right of the fixation dot, at a distance of 9.5° from the center. Each box contained overlapping M and N white letters. In the cue display, each placeholder box was surrounded by four white dots (0.4° wide), except for the cued box, which was surrounded by four red dots [CIE(0.621, 0.345), 39.7 cd/m^2^]. In the target display, two randomly chosen placeholder boxes contained exclusively the letter M, and the other two exclusively the letter N. One of these four letters was red, defining the target letter, whereas the other letters were white. When fixated on the central dot, it was possible to discern the placeholder boxes but not their contents, so the participants needed to make an eye movement to identify the target letter. In the onset condition, the target display included one extra white placeholder, which also contained a white distractor letter (equiprobably M or N). This extra box was placed equiprobably in the middle of any two of the other boxes, at an equal distance from fixation. In the remove condition, the final display consisted of only the placeholder box with the red target letter inside.

#### Design and procedure

The design included three within-subjects factors: Cue Validity (valid, invalid), Onset Presence (absent, present), and Distractor Removal (not remove, remove). The red cue was valid only at chance level (thus correctly predicting the location of the target on 25 % of the trials), and was therefore uninformative about the location of the upcoming target. On 50 % of the trials, an extra placeholder box appeared (i.e., the onset-present condition). On half of the trials, all objects in the target display except for the placeholder box with the target letter turned off during the execution of the first saccade (i.e., the *remove* condition). On the other half of the trials, the distractors remained unchanged until the participant responded (i.e., the *not*-*remove* condition). All three factors were randomly presented within eight blocks of 96 trials each. Participants started with an additional practice block of 96 trials.

Participants were tested in a single session lasting approximately 75 min. They were instructed to look for a red letter, M or N, inside one of the placeholder boxes, and to respond as quickly as possible to the identity of this letter while maintaining high accuracy. Participants were furthermore instructed to fixate their eyes on the central dot at the start of every trial until the cue display appeared. From this moment, eye movements were allowed. Participants were also told that the red cue was uninformative and that the onset never contained the target letter.

As is shown in Fig. 1, at the start of each trial, the fixation dot briefly flashed for 100 ms, and, after another 1,000 ms, the cue display was presented for 50 ms. After an interstimulus interval of 100 ms, the target display appeared, which contained an extra placeholder box with a white M or N (i.e., the onset) in the onset-present condition. In the remove condition, all distractors disappeared after the eyetracker registered that participants had started the execution of the first saccade; in the not-remove condition, the target display was unchanged until the manual response. A saccade was defined as an eye movement of which the acceleration was greater than 9500°/s^2^ and the velocity exceeded 35°/s. A saccade was assigned to a particular object if the angular deviation between the center of the object and the saccade’s endpoint was less than 11.25° (corresponding to half the angular distance between an onset and its adjacent objects) along the imaginary circle on which the objects were positioned. The latencies of first saccades and manual responses were time-locked to the onset of the target display.

Upon identification of the target, participants pressed the “M” key if the target letter was an M and the “N” key if it was an N. The target display was replaced by the fixation display after the manual response or after a maximum response interval of 2,000 ms had expired. In the case of a wrong response, the fixation dot turned red and simultaneously a buzzer sounded for 250 ms. A 1,000-ms intertrial interval, showing the fixation display, started immediately after a correct response/response omission or after the error response feedback.

### Results

Practice trials were discarded and trials with incorrect keypresses were removed (5 %), as were trials with RTs below or above 2.5 *SD*s from the participant’s condition mean (another 2 %). Trials on which no saccade to the target item was detected were also removed (<1 %), as were trials with saccades to locations other than those of the cue, target, or onset (<1 %).

#### Manual response data

**Fig. 2:**
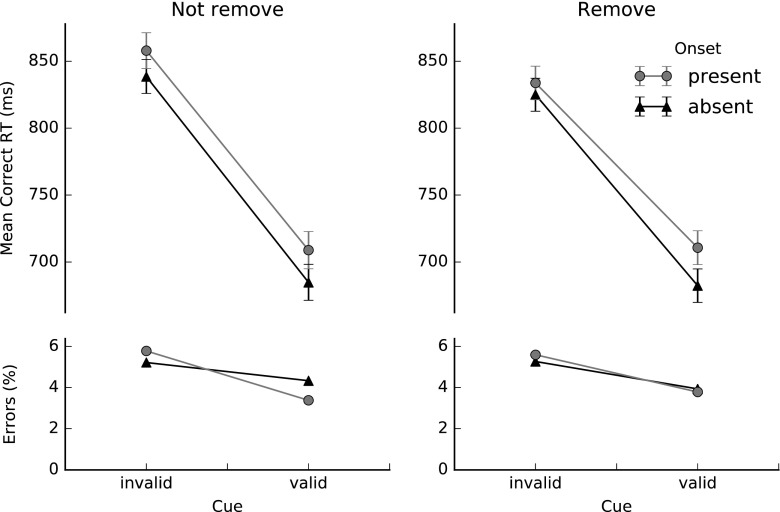
Mean manual response times (RTs) and error percentages in Experiment 1 for valid and invalid cues, with and without the presence of an abrupt onset, in the not-remove and remove conditions. The error bars represent ±1 *SE* of the condition mean

Figure 2 shows the mean manual correct RTs and accuracy scores in the not-remove and remove conditions for valid and invalid cues, with and without the presence of an abrupt onset. A repeated measures analysis of variance (ANOVA) was conducted with Cue Validity (valid or invalid), Onset Presence (absent or present), and Distractor Removal (not remove, remove) as within-subjects factors. Most importantly, we observed a significant three-way interaction between cue validity, onset presence, and distractor removal, *F*(1, 23) = 4.72, *p* = .041, *η*_p_^2^ = .17, indicating that the relation between cue validity and onset presence differed for the remove and not-remove conditions.

To specify this interaction, separate two-way ANOVAs were conducted on the data of the remove and not-remove conditions. In the not-remove condition, we found a near-perfect additivity between the effects of cue validity and onset presence, *F*(1, 23) < 1, *p* = .422, *η*_p_^2^ = .03. The effect of cue validity was 149 ms in the onset-present condition, and 154 ms in the onset-absent condition. By contrast, in the remove condition, a two-way ANOVA revealed a highly significant underadditive interaction between cue validity and onset presence, *F*(1, 23) = 18.89, *p* < .001, *η*_p_^2^ = .45, indicating that the effect of cue validity was smaller when the onset was present (123 ms) than when it was absent (143 ms).

Furthermore, the ANOVA showed main effects of cue validity and onset presence in both the remove and not-remove conditions. In the not-remove condition, participants were on average 151 ms faster when the cue was valid than when it was not valid, *F*(1, 23) = 264.78, *p* < .001, *η*_p_^2^ = .92, and 22 ms faster when the onset was absent than when it was present, *F*(1, 23) = 40.51, *p* < .001, *η*_p_^2^ = .64. In the remove condition, again participants were faster (mean difference = 133 ms) when the cue was valid than when it was invalid, *F*(1, 23) = 278.49, *p* < .001, *η*_p_^2^ = .92, and when the onset was absent than when it was present (mean difference = 19 ms), *F*(1, 23) = 41.48, *p* < .001, *η*_p_^2^ = .64.

The error rates for the various conditions were all below 7 % (see also Fig. 2). An ANOVA revealed a main effect of cue validity, indicating that participants made significantly more errors in the invalid-cue condition (an average error rate of 5 %) than in the valid-cue condition (an average error rate of 4 %), *F*(1, 23) = 8.54, *p* = .008, *η*_p_^2^ = .27. There were no significant differences in error rates between the not-remove and remove conditions, *F* < 1, and between the onset-present and onset-absent conditions, *F*(1, 23) < 1, *p* = .882. We also observed no significant interactions.

**Fig. 3:**
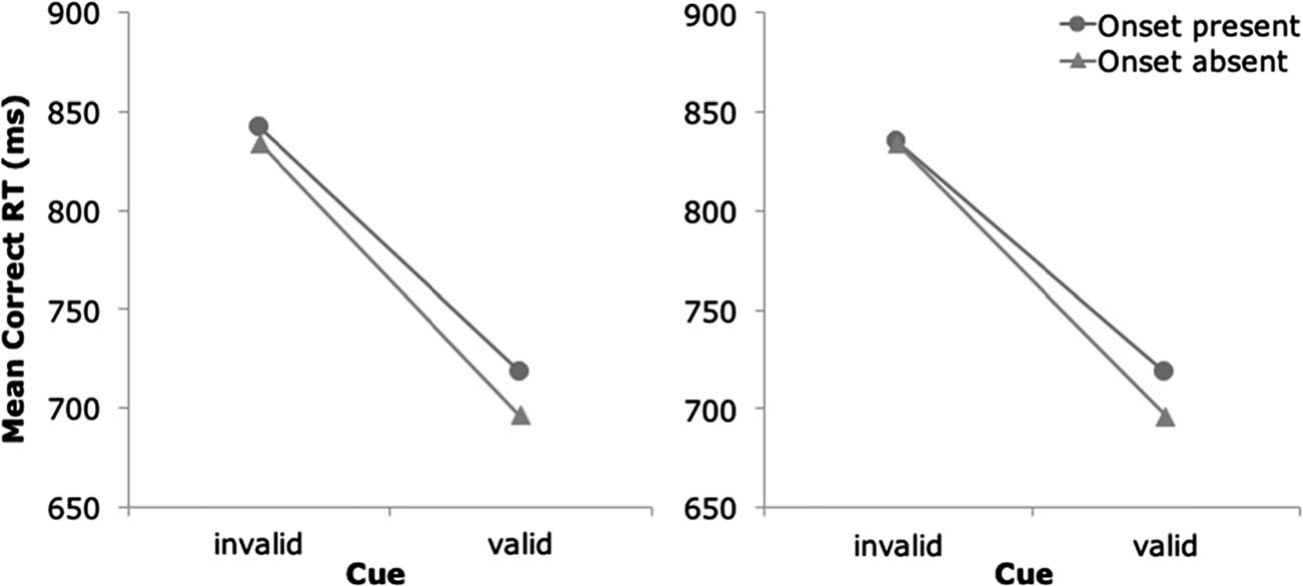
Comparison of the mean correct RT patterns in the remove (right) and not-remove (left) conditions when trials with three-saccade trajectories were removed from the data set

According to the one-process view of Schreij et al. (2014), the three-way interaction in the RT data results from a reduction of three-saccade trajectories in the remove condition relative to the not-remove condition. To examine whether this was indeed the case, we analyzed the RT data of Experiment 1 after discarding all trials on which three-saccade trajectories occurred. This led to the removal of 6.4 % of all trials in the invalid-cue, onset-present condition (8.1 % of the trials in the not-remove condition, 4.7 % of the trials in the remove condition), whereas no trials were removed in the other conditions. The resulting RT data are shown in Fig. 3. Clearly, after removal of the three-saccade trajectories, an underadditive interaction between cue validity and onset presence was observed, independent of removal condition. This impression was confirmed by a three-factor ANOVA with cue validity, onset presence, and removal condition as within-subjects variables. The two-way interaction between cue validity and onset presence was highly significant, *F*(1, 23) = 16.81, *p* < .001, *η*_p_^2^ = .42, whereas the three-way interaction was not, *F* < 1. The results of this analysis confirm that three-saccade trajectories may prevent the emergence of the underadditive interaction in the RT data.

#### Saccade data

**Fig. 4:**
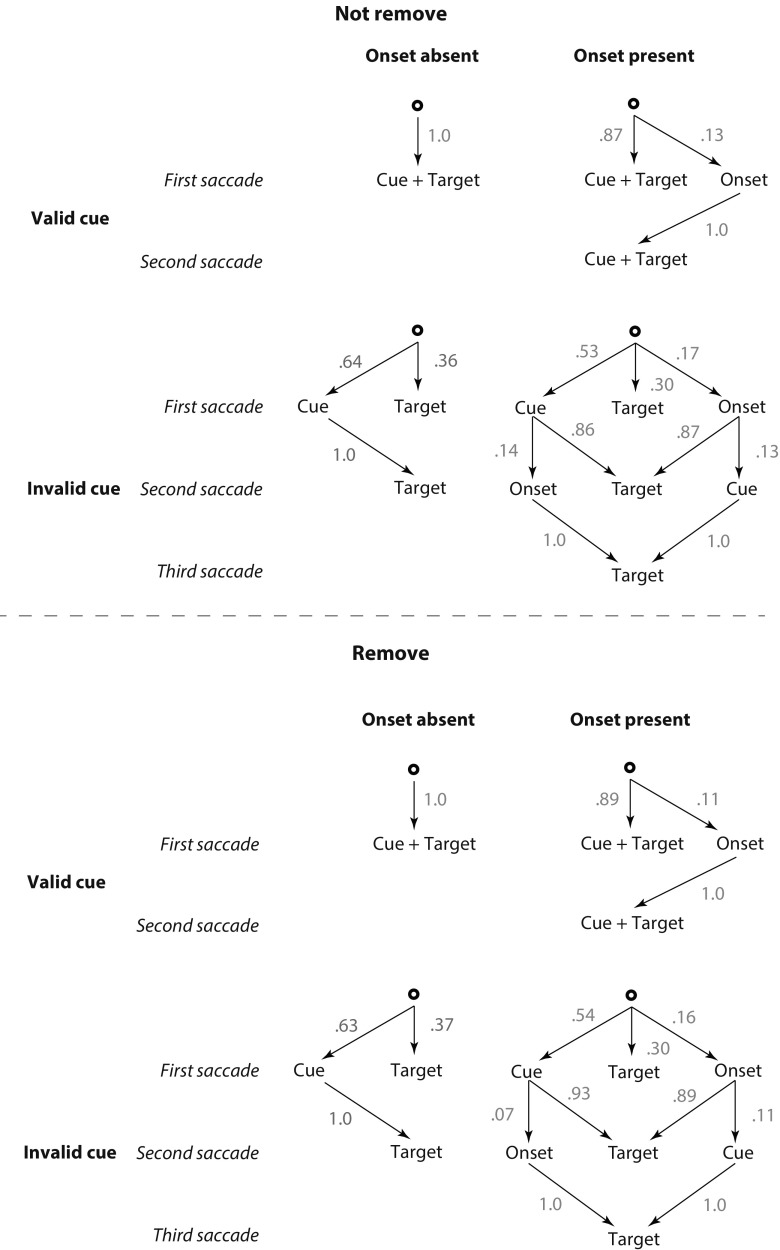
Conditional probabilities of first, second, and third saccades to each item of interest in the visual field, for all possible combinations of cue validity, onset presence, and distractor removal. Saccades to locations other than those of the cue, target, or onset rarely occurred and are therefore not taken into account

Figure 4 shows the possible paths the eyes took before arriving at the target location, along with the conditional probabilities for these paths. One can notice that there is hardly any difference between the probability distributions in the remove and not-remove conditions, except for the proportions of three-saccade trajectories in the onset-present, invalid-cue condition. In the not-remove condition, 14 % of the saccades visited the onset after having initially been captured by the cue, whereas in the remove condition, only 7 % of the second saccades still visited the onset’s old position (even though it was no longer visible), *t*(23) = 4.30, *p* < .001. The remove condition also led to a slight, 2 % reduction of three-saccade trajectories when the eyes were initially captured by the onset, but this difference failed to reach significance, *t*(23) = 0.859, *p* = .377 (as did all other paired comparisons between corresponding saccade proportions in the remove and not-remove conditions). In all, although the onset and the cue still attracted some second saccades in the remove condition, this happened less often than in the not-remove condition, meaning that our new manipulation (distractor removal) largely succeeded.

A second finding that should be noted in the saccade data pertains to the proportions of first saccades toward the different objects in the display. Replicating earlier findings by Schreij et al. (2014), the onset attracted a substantial percentage of first saccades to its location (14 %, averaged across the distractor removal conditions), which led to a reduced percentage of first saccades to the invalid cue (from 64 % to 54 % in the onset-absent and onset-present conditions, respectively), *t*(23) = 7.36, *p* < .001. This finding indicates that both the onset and the cue vied for attention, so the presence of an onset led to a reduction of saccades toward the invalid cue.

**Fig. 5:**
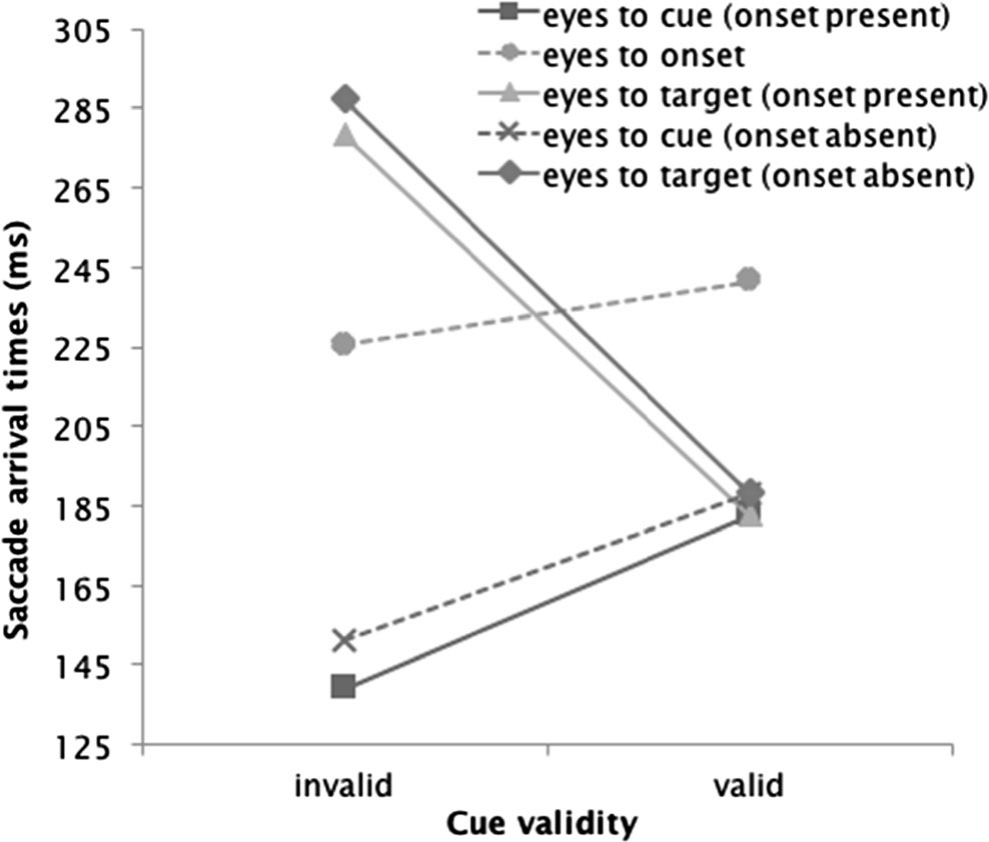
Arrival times of first saccades in correct trials at each relevant object on the display, averaged over the remove and not-remove conditions

Finally, to zoom in on the processing dynamics preceding the first saccade, we analyzed first-saccade latencies conditional on the endpoint of the saccade (i.e., either the target, the cue, or the onset) as a function of cue validity and onset presence. As Fig. 5 shows, the shortest saccade latencies were observed for eye movements to the cue, which reflects that the cue was presented 150 ms prior to the target display, whereas latencies were all measured relative to the onset of the target display. Also note that, in the valid-cue condition, the cue and the target shared the same location, which explains why the first-saccade latencies were identical for saccades to the valid cue and the target.

Our main reason to study first-saccade latencies concerned the influence of onset presence. According to the two-process account (Folk & Remington, 1998), the alleged filtering process has more work to do when an onset is present than when it is absent, which should slow down the first saccade to the cue or the target. By contrast, according to the one-process account (Schreij et al., 2014), the presence of an onset merely increases competition among potentially relevant saccade locations. Although this enhanced competition should have an effect on where the eyes go (as is shown by Fig. 4), it should not slow down the first saccade to either the target or the cue. As Fig. 5 shows, the data clearly confirm the predictions of the one-process account: First saccades to the target or the cue were even slightly faster, not slower, when an onset was present than when it was absent.

An ANOVA on the latencies of saccades going to the cue location, with repeated measures for cue validity and onset presence, yielded a main effect of cue validity, *F*(1, 23) = 66.86, *p* < .001, *η*_p_^2^ = .74, indicating that latencies were 40 ms shorter in the invalid-cue than in the valid-cue condition, and a main effect of onset presence, *F*(1, 23) = 16.02, *p* = .001, *η*_p_^2^ = .41, indicating that latencies were 9 ms shorter in the onset-present than in the onset-absent condition, with no significant interaction, *F*(1, 23) = 2.14, *p* = .164. The corresponding ANOVA on the latencies of saccades going to the target yielded main effects of cue validity, *F*(1, 23) = 174.80, *p* < .001, *η*_p_^2^ = .88, indicating that latencies were 98 ms shorter in the valid-cue than in the invalid-cue condition, and of onset presence, *F*(1, 23) = 6.42, *p* = .019, *η*_p_^2^ = .22, indicating that latencies were 6 ms shorter in the onset-present than in the onset-absent condition, with no interaction, *F* < 1. Finally, for saccades going to the onset (in the onset-present condition), the ANOVA revealed a significant effect of cue validity, *F*(1, 23) = 4.78, *p* = .039, *η*_p_^2^ = .17, indicating that latencies were 19 ms shorter in the invalid-cue than in the valid-cue condition.

### Discussion

When all distractor items remained on the screen until the end of the trial (the not-remove condition), the effects of cue validity and onset presence on manual RTs were found to be additive, just as in previous studies (Folk et al., 2009; Schreij et al., 2014; Schreij et al., 2008). However, when distractor items were removed from the display during the first saccade (the remove condition), an underadditive pattern emerged. These findings constitute strong evidence against the two-stage account proposed by Folk and colleagues (Folk & Remington, 1998; Folk et al., 2009) and in favor of the one-process account proposed by Schreij et al. (2014).

According to the two-stage account, a nonspatial filtering operation, selectively influenced by onset presence, precedes attentional orienting, selectively influenced by cue validity. According to the classic additive-factors logic (Sternberg, 1969, 2001), onset presence and cue validity should therefore reveal additive effects, as was observed in the not-remove condition of the present study as well as in all previous studies. Crucially, though, a two-stage solution also requires that an additive relationship between two factors should persist, regardless of the level of any third factor that is added to the design (Ridderinkhof & Van der Molen, 1995; Sanders, 1990; Sternberg, 1969, 1998). The present finding that the additive relationship between onset presence and cue validity in the not-remove condition changed to an interaction in the remove condition is a clear violation of this “stage robustness” principle. A stage solution of our findings is therefore untenable.

According to the alternative, one-process view proposed by Schreij et al. (2014), a noncontingent onset and a contingent cue both influence the same spatial orienting process. The reason that these factors do not interact on manual mean RTs in the not-remove condition is that, during the preceding eye movements, the presentation of an onset has an early and a late modifying influence on the cue validity effect, in opposite directions. Its early influence concerns a reduction of the proportion of first saccades going to the invalid cue, which by itself should lead to a reduction of the cue validity effect on manual RTs; its late influence concerns the emergence of three-saccade trajectories in the invalid-cue condition, which by itself should lead to an increase of the cue validity effect on manual RTs. To the extent that the early and late influences balance out, onset presence and cue validity will have additive effects on manual mean RTs. These dynamics, first observed by Schreij et al. (2014), were replicated in the not-remove condition of the present study. The critical new finding is the observation of a reduction of three-saccade trajectories in the remove condition, which in turn led to an underadditive interaction on manual mean RTs. This finding is fully consistent with the interpretation proposed by Schreij et al. (2014), and constitutes further evidence that an onset influences a spatial orienting mechanism rather than a nonspatial filtering operation.

It is furthermore noteworthy that three-saccade trajectories were not completely absent in the remove condition (see Fig. 4). In fact, as compared to the not-remove condition, there was no significant reduction of three-saccade trajectories in the remove condition when the eyes were initially captured by the onset. This reduction was substantial (by a factor of 2) when the eyes were initially captured by the invalid cue, but even here the onset preserved its potency to attract attention after it had disappeared during an intervening saccade. As a result, the second saccade occasionally arrived at the empty spot previously occupied by the onset. This finding just underscores the onset’s potency to capture attention (see Godijn & Kramer, 2008, for similar findings).

Although our remove manipulation did not abolish all three-saccade trajectories, the reduction turned out to be sufficient to turn the additive effects of cue validity and onset presence on mean RTs into an underadditive interaction. In further support of this mechanism, an additional analysis revealed that after discarding the trials on which three-saccade trajectories occurred, a powerful underadditive interaction emerged, regardless of removal condition (Fig. 3). This underscores that the normally observed additive effects of these factors stem from a delicate balance of two counteracting influences. Reduction of the late influence (i.e., competition for attention after the first saccade, which occasionally leads to three-saccade trajectories), whether experimentally (in the remove condition) or by post-hoc selection of trials (as in Fig. 3), leads to an expression of the first influence on mean RTs. The first influence reflects that the onset and the cue vie for spatial attention from the moment the target display is presented, as is claimed by the one-process account.

Finally, if the effect of onset presence on mean RTs reflects filtering costs, as is proposed by the two-process account (Folk & Remington, 1998; Folk et al., 2009), one would predict that first-saccade latencies to the cue or target would be longer in the onset-present than in the onset-absent condition. Schreij et al. (2014) failed to confirm this prediction, by showing a null effect of onset presence on first-saccade latencies, whereas the present data even showed a reversed effect: First-saccade latencies to either the cue or the target were slightly shorter in the onset-present than in the onset-absent condition. From the perspective of the one-process account, this finding is not problematic. In a competitive environment, adding an onset to the target display may snatch off some long-latency saccades that would have otherwise gone to the cue or target, leading to a reduction of the mean first-saccade latency to the cue or target. Thus, whereas the “reversed” onset effect on first-saccade latencies is problematic for the two-process view, it can easily be reconciled with the one-process account.^1^

To summarize, all facets of the present data set, running from the first saccade all the way to the manual response, favor the one-process account (Schreij et al., 2014) over the two-process account (Folk & Remington, 1998; Folk et al., 2009). First, initial saccades to either the cue or the target stimulus were not slowed down by the presence of an onset, but even slightly sped up, reflecting competition for spatial attention. Second, an onset attracted first saccades to its location at the cost of first saccades going to the target or the cue, again showing that it joined the competition for spatial attention. Third, in the condition with an invalid cue, an onset occasionally gave rise to reaching the target after three saccades, showing that it may attract spatial attention even after the eyes had initially been captured by an invalid cue. Fourth, removal of the distractors after the first saccade led to a reduction of three-saccade trajectories, presumably reflecting reduced competition for spatial attention. Fifth, the reduction of three-saccade trajectories gave rise to an underadditive interaction on manual RTs, which has long been considered the critical prediction for a one-process account in which the onset and cue vie for spatial attention. Sixth, removal of the distractors after the first saccade changed the additive effects of onset presence and cue validity on manual RTs into an underadditive interaction—a pattern predicted by the one-process account but fundamentally inconsistent with a two-stage solution (i.e., a violation of stage robustness).

## Experiment 2

In Experiment 1, participants had to move their eyes through the search field to be able to perform the task, because the individual items would otherwise not be discriminable, due to their large retinal eccentricity. We thus demonstrated that the underadditive pattern between onset presence and cue validity can occur for overt attention shifts. This leaves open the logical possibility that this effect is bound to the occurrence of eye movements, so it would not generalize to paradigms involving covert shifts of attention. If so, this would severely limit the generality of our findings.

The goal of Experiment 2 was therefore to investigate whether the underadditive pattern can also be found when attention is shifted covertly, thus without making eye movements to the location that is attended. To disentangle the effects of eye movements from pure shifts of spatial attention, we adapted our paradigm to a conventional attentional-capture paradigm in which the target is searched while the eyes remain centrally fixated. For this purpose, we adjusted the layout of the displays such that all letters could be identified from fixation. To implement the remove condition, we faced the problem that we could not rely on overt eye movements, contingent on which distractors were turned off in Experiment 1. To deal with this problem, we assumed that the latency of the first covert shift in visual attention in the attentional-capture paradigm is proportional to the latency of the first saccade in the oculomotor-capture paradigm. In Experiment 1, the ratio of the mean first-saccade latency to the mean manual RT was very roughly .20. Furthermore, since the anticipated mean RT in the attentional-capture version of our paradigm was approximately 0.5 s (Schreij et al., 2008; Schreij et al., 2010a, 2010b), we fixed the exposure duration of the distractors in the remove condition at 100 ms. Of course, apart from being very coarse, this estimate fails to incorporate any sources of both between- and within-subjects variability. To compensate for the inevitable loss of power, we decided to involve a relatively large sample of participants in Experiment 2.

To the extent that Experiment 2 yielded findings similar to those of Experiment 1, it would suggest that shared underlying mechanisms are at work. In particular, if we again observed that additive effects of onset presence and cue validity in the not-remove condition turned into an underadditive interaction in the remove condition, this would strongly suggest that our manipulations influenced covert attentional orienting, not the mere propensity of making eye movements.

### Method

#### Participants

A new sample of 40 students (13 men, 27 women, between 19 and 30 years of age, *M* = 25.5) at VU University of Amsterdam participated in this study. All reported normal or corrected-to-normal vision and no color blindness. They signed an informed consent.

#### Procedure

The apparatus and experimental design were the same as in Experiment 1, with the exception that the eyetracker was no longer used. Stimuli were presented on a 22-in. Samsung 120-Hz LCD screen with a resolution of 1,680 × 1,050 pixels and at a distance of 80 cm from the participant. The stimuli were enlarged and positioned closer together so that they were easily discernable from fixation. Participants were instructed to perform the task while keeping their eyes fixated on the central dot. Even though it is possible that participants did not follow this instruction and did make eye movements, we deemed this highly unlikely, because such a strategy would only hamper their performance. The placeholder boxes were 1.67° wide and placed at a distance of 6.11° (center to center) from the fixation dot. Each cue dot had a radius of 0.26°. The procedure was identical to that of Experiment 1, with the exception that the disappearance of the irrelevant items in the remove condition was no longer contingent on the first saccade of the participant, but instead occurred after a fixed interval of 100 ms after presentation of the target display.

### Results

**Fig. 6:**
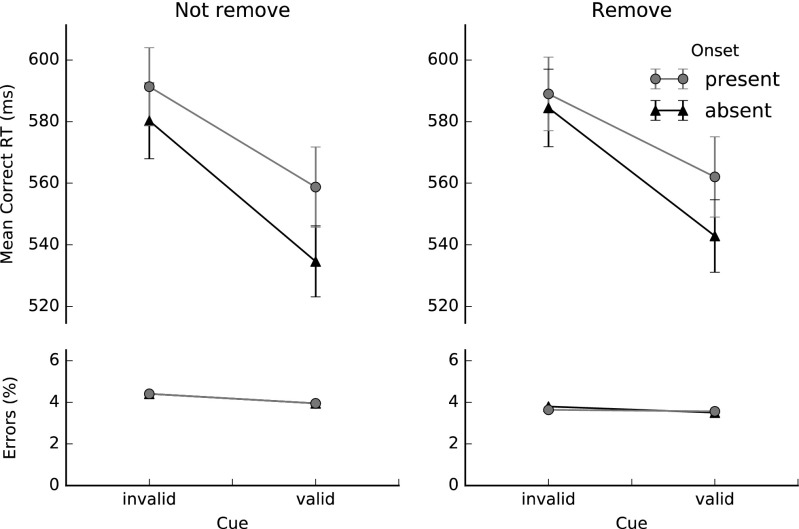
Mean manual response times (RTs) and error percentages in Experiment 2 for valid and invalid cues, with and without the presence of an abrupt onset, in the not-remove and remove conditions. Note that, due to the equal error percentages for both accuracy series in the not-remove condition, only one line is discernible. The error bars represent ±1 *SE* of the condition mean

One participant was excluded from the data set because of a malfunctioning apparatus, and another one because his overall accuracy was below 80 %. All manual responses were produced within the maximum time limit of 2,000 ms. We discarded practice trials and trials on which the given response was incorrect (4.0 %). For each participant, trials on which RTs were faster or slower than 2.5 *SD*s from the participant’s condition mean were also removed (another 2.7 %). Figure 6 shows the mean correct manual RTs as a function of cue validity and onset presence, separately for the not-remove and remove conditions.

The manual RTs were submitted to a repeated measures ANOVA with Cue Validity (invalid, valid), Onset Presence (absent, present), and Distractor Removal (not remove, remove) as factors. Participants’ manual responses were significantly slower when either the cue was invalid (by 37 ms) or an onset distractor was present (by 18 ms), *F*(1, 37) = 188.58, *p* < .001, *η*_p_^2^ = .84, and *F*(1, 37) = 41.99, *p* < .001, *η*_p_^2^ = .53, respectively. The interaction between cue validity and onset presence was also significant, *F*(1, 37) = 8.46, *p* < .005, *η*_p_^2^ = .19. When an onset distractor appeared, the facilitating effect of a valid cue (30 ms) was reduced as compared to when there was no onset (44 ms). Distractor removal had no main effect, *F*(1, 37) = 2.87, *p* = .098, *η*_p_^2^ = .07, nor did it interact with cue validity, *F*(1, 37) = 2.51, *p* = .121, *η*_p_^2^ = .06, or onset presence, *F*(1, 37) = 2.41, *p* = .129, *η*_p_^2^ = .06. The three-way interaction among all factors also failed to reach significance, *F*(1, 37) < 1, *p* = .841, *η*_p_^2^ = .001.

Although the three-way interaction was far from significant, we conducted two separate two-way ANOVAs on the data of the remove and not-remove conditions to maximize comparability with the results of Experiment 1. The interaction between cue validity and onset presence only approached significance in the “classic” situation in which all stimuli remained on screen, *F*(1, 37) = 3.33, *p* = .076, *η*_p_^2^ = .08, but attained significance in the remove condition, when all items but the target disappeared after a short period, *F*(1, 37) = 8.27, *p* = .006, *η*_p_^2^ = .18.

The error rates were all below 5 %. An ANOVA with Cue Validity, Onset Presence, and Distractor Removal as factors revealed only a main effect of distractor removal, *F*(1, 37) = 4.12, *p* = .049, *η*_p_^2^ = .10: Participants responded less accurately when all stimuli remained on the screen.

### Discussion

The data of Experiment 2 revealed a clear underadditive interaction between cue validity and onset presence, but, unlike the findings of Experiment 1, this interaction was not modified by distractor removal. The underadditive interaction in the remove condition replicated the corresponding finding of Experiment 1 and was predicted by the one-process account (Schreij et al., 2014). The finding that this interaction approached significance in the not-remove condition was more surprising, given that previous studies, including the not-remove condition of Experiment 1, had invariably revealed fairly clean additive effects of cue validity and onset presence (Schreij et al., 2014; Schreij et al., 2008; Schreij et al., 2010a, 2010b; Wu et al., 2014). It is not fully clear what might account for this discrepancy. One possibility relates to the fact that the remove and not-remove conditions were presented in mixed blocks of trials. It is conceivable that through strategic adjustments or intertrial dynamics (see, e.g., Los, 1996), the remove condition somehow contributed to the two-way interaction in the not-remove condition. To explain why a similar carryover effect did not occur in Experiment 1, one might speculate that the nondisruptive nature of distractor removal during the saccadic eye movement somehow prevented its manifestation.

Whatever the virtue of this speculation, it is important to recall that, according to the one-process account (Schreij et al., 2014), the finding of additive effects of cue validity and onset presence relies on a delicate balance between two counteracting influences of the onset. On the one hand, the onset decreases the proportion of first saccades to the cue location, thereby reducing the effect of cue validity; on the other hand, the onset increases the proportion of three-saccade trajectories in the case of an invalid cue, thereby increasing the effect of cue validity. As is shown by the data of the not-remove condition of Experiment 1, these influences balanced out (see also Schreij et al., 2014), thus leading to additive effects of cue validity and onset presence on manual RTs. The upshot from this view is that even a slight disruption of this delicate balance will turn the additive pattern of effects into an (underadditive) interaction, especially when the sample size is large. In view of this, finding the underadditive interaction approaching significance in the not-remove condition of Experiment 2 is not very remarkable.

More generally, the most important finding of Experiment 2 is the very occurrence of the underadditive interaction, regardless of whether one considers it separately for the levels of distractor removal or averaged across them. This finding challenges the view that the contingent cue and the noncontingent onset stimulus influence RTs at different processing stages (Folk & Remington, 1998; Folk et al., 2009). According to this account, these variables influence two consecutive processing stages (spatial attentional orienting and nonspatial filtering, respectively), and should therefore have additive effects, regardless of the level of whatever additional factor in the design (Sternberg, 1969, 1998, 2001). By contrast, the underadditive interaction is consistent with the one-process account that both stimuli influence the same underlying mechanism (Schreij et al., 2014).

What is the nature of this mechanism? Since participants performed the task of Experiment 2 without making eye movements, it is highly unlikely that a mechanism involving the programming or production of eye movements was responsible for the underadditive interaction between cue validity and onset presence—a possibility the data of Experiment 1 still left open. Instead, it is more reasonable to assume that mechanisms at the level of spatial attention were the driving force behind the observed underadditivity. The evidence of a tight coupling between attention and eye movements (Hulleman & Olivers, 2015; Rizzolatti et al., 1987) further suggests the intriguing possibility that in Experiment 2, attention followed similar pathways across the stimulus displays to those followed by the eyes in Experiment 1.

## General discussion

Using the same paradigm as Schreij et al. (2014), we found additive effects of cue validity and onset presence when all distractors remained on screen until response in Experiment 1, corroborating previous research (Schreij et al., 2014; Schreij et al., 2008; Schreij et al., 2010a, 2010b; Wu et al., 2014). However, when removing all distractor items from the display during the first saccade, an underadditive relationship between cue validity and onset presence emerged. This study was the first to reveal such an underadditive relationship. The transition from additive effects toward an underadditive interaction is a clear violation of stage robustness (Sternberg, 1969, 1998), and therefore constitutes evidence against the two-stage model proposed by Folk et al. (2009). In addition, inspection of the eye movement data revealed that this transition was attributable to a reduction of three-saccade trajectories in the remove condition relative to the not-remove condition, consistent with the one-process account of Schreij et al. (2014). According to this account, the contingent cue and the onset vie for attentional control, and keep on doing so even after the first eye movement. Thus, by removing the distractor elements during the first saccade, competition was reduced later on, which led to a reduction of three-saccade trajectories and ultimately changed the additive pattern into an underadditive one.

The findings of Experiment 2 do not, by themselves, allow inferences that are as clear-cut as those of Experiment 1, for the obvious reason that covert shifts of attention cannot be tracked in the same way as overt eye movements. As a result, we did not have access to trial-by-trial information regarding attentional trajectories, and we therefore could not synchronize the removal of the distractor elements with the onset of the first shift of attention. Indeed, our estimate of this moment was coarse and did not incorporate any intertrial and interindividual variability. Finally, since the removal of the distractors was not shielded by a saccadic eye movement, this event may have been somewhat disruptive in general. Despite all of these inevitable limitations, the results of Experiment 2 are still noteworthy because they show, for the first time in the attentional-capture paradigm, an underadditive interaction between cue validity and onset presence.

This finding is important because it is difficult to accommodate with the two-stage account (Folk & Remington, 1998; Folk et al., 2009). If onset presence selectively influences the duration of a nonspatial filtering stage, whereas cue validity influences the duration of a later attentional orienting stage, the effects of these variables should be additive according to standard additive-factors logic (Sternberg, 1969, 1998, 2001). Therefore, to reconcile the underadditive interaction in the remove condition with the two-stage account, one has to make additional assumptions. One attempt in this respect starts from the idea that, when the onset is absent, the filtering stage would be over quickly, such that the subsequent attentional orienting stage would likely be strongly affected by the still-present distractor elements. Conversely, in the presence of the onset, the duration of the filtering stage would be extended, which would delay the beginning of the selection stage, and hence increase the probability that the distractor elements would be gone when attentional orienting started. As a result, in the remove condition, a stronger effect of cue validity would be expected in the onset-absent condition than in the onset-present condition, thus explaining the underadditive relationship while maintaining the integrity of the two-stage architecture.^2^

Whereas this account could explain the results of Experiment 2, it clearly falls short of accounting for the results of Experiment 1. In the remove condition of this experiment, the changes in the display were triggered by the first saccade of the participant, and were therefore tightly coupled to the completion of the putative filtering process. Moreover, the latency of the first saccade to any object other than the onset was even slightly sped up by the presence of an onset, rather than delayed (Fig. 5). If the latency of the eye movements to the target or the invalid cue is not delayed by onset presence, there is little ground to believe that a filtering stage takes longer in the onset-present than in the onset-absent condition.

In contrast, when considered across experiments, the present findings draw a very consistent picture from the perspective of the one-process account (Schreij et al., 2014). According to this account, both the contingent cue and the noncontingent onset influence attentional orienting en route to the target stimulus. The oculomotor-capture paradigm provides a detailed view of the underlying dynamics and strongly suggests that both stimuli vie for control over the oculomotor system. Although the results of the attentional-capture paradigm are inevitably less revealing in this respect, the behavioral results are generally consistent with those obtained in the oculomotor paradigm. This consistency reinforces the general principle of a tight coupling between eye movements and spatial attention (Rizzolatti & Craighero, 1998; Rizzolatti et al., 1987) and the idea that eye movement trajectories can be profitably used as a model for covert attentional orienting (Hulleman & Olivers, 2015; Schreij et al., 2014).

An intriguing question is to what extent our conclusion might generalize to other paradigms that have examined the combined effects of stimulus-driven and goal-driven attention. For instance, in the studies of Juola, Koshino, and Warner (1995) and Berger, Henik, and Rafal (2005), a central arrow cue was used to specify with a probability well above chance the location of the impending target stimulus, thereby inducing goal-driven spatial orienting. In addition, an uninformative peripheral cue briefly flashed before target onset, inducing stimulus-driven spatial orienting. Both studies revealed additive effects of the validity of these cues, which led the authors to conclude that the two processes operate independently in controlling attention. However, the findings of the present study suggest the alternative interpretation that this additivity, again, is the result of averaging over different trajectories of covert attention occurring within each condition. Whereas on some trials contingent capture may have driven attention, on other trials attention could have been driven by the exogenous cue. Thus, the additive results reported in these studies do not necessarily indicate that both processes function completely apart from each other.

In conclusion, the present study provides strong support for the view that both the validity of a contingent cue and the presence of a noncontingent onset influence a common spatial orienting mechanism. Whereas previous studies had supported this view through analyses of the spatiotemporal trajectories of saccades, the present study revealed this common orienting mechanism by showing, for the first time, an underadditive interaction regarding manual RTs. Moreover, the present study showed that the underadditive interaction can also be observed when eye movements are precluded, thereby revealing the true attentional nature of the underlying mechanism.

## Footnotes

1 A conspicuous feature of the data in Fig. 5 is that first saccades to the valid cue/target location are much slower than first saccades to the invalid cue, and much faster than first saccades to the target location in the invalid-cue condition. Schreij et al. (2014) obtained a very similar pattern and modeled the saccade latencies in the valid cue/target condition as a mixture of the fast and slow saccades obtained in the other two conditions, deriving the mixture proportion from the relative first-saccade frequencies (i.e., the present Fig. 4). The mixture model fitted the data remarkably well, accounting for more than 99 % of the variance. Since this aspect of the data goes well beyond the purpose of the present article, we decided not to repeat this analysis here.

2 We thank Kyle Cave for suggesting this alternative account.
